# The New Outlook

**Published:** 1920-08-28

**Authors:** 


### SANITARY SCIENCE AND PREVENTIVE MEDICINE.

### The New Outlook.

Many aspects of the preventive-medicine problem have been authoritatively dealt with in the lectures and official addresses of recent times, and each has had considerable fascination for those who would really keep abreast of modern medical thought. But one particularly has lately caught our attention, and that because the technical subject with which it deals tends often to be but half appreciated in its true and vital bearing upon the science of maintaining a healthy nation. Sanitary science has been a gravely misunderstood—often misrepresented—term, and Colonel Sir Robert Firth, K.B.E., C.B., F.R.C.S., rendered a valuable service in helping us to pick up dropped threads and re-orientate ourselves in this particular aspect of preventive medicine when, under the above title, he delivered his Presidential address at the recent Birmingham Congress.

It has now been fairly well rubbed in that preventive medicine which restricts its scope to environmental questions and the amenities of external sanitation is a conception now once and for all discarded. Doubtless if, at the moment, we select any particular populous district we should find many residues of the old cumbersome anomalies, which lead to such hopeless confusion and overlapping; but whatever the critics may say, these things should gradually disappear, and the formation of a Ministry of Health promises well for simplification and unification in the all-important questions of general sanitation, housing, epidemiology, infectious disease, maternity and infant welfare, health insurance, tuberculosis and venereal disease, with co-ordination of the hygienic activities of other departments such as the Board of Education for the school child, the Home Office for industrial hygiene, and the Board of Agriculture for food control. The future should at least bring that unified official administration which can—if only it is truly carried out with efficiency as its ideal—produce the correct setting in which "preventive medicine as the chief handmaiden of the new humanity" can make her entrance.

Among all the cries for reconstruction we must not forget that sanitary science, in its wider sense, has achieved much in the past. Astonishing victories have been gained in the past fifty years. Vast sections of the population live relatively healthy lives; at all ages there has been a reduction of the death-rate; the expectation of life at birth has risen in the case of males from 41 to 51 years, and in that of females from 44 to 55 years; the infant-mortality rate is well below the 10 per cent. line; we suffer a relatively light burden from some epidemic and infectious diseases, and many gross forms of disease have practically disappeared, while whole groups of tropical diseases have come, or are coming, under control. On the other hand, we still have a steadily falling birth-rate, a still unnecessary loss of life in infancy and before birth. Nearly 100,000 fresh cases of tuberculosis occur annually; a similar number of diphtheria and scarlet fever cases, and upwards of a million cases of measles. And so might we run on through all the proved percentage deficiencies of the school children and the unbelievable amount of unfitness which the recruiting figures showed. Gradually, as we build up a national health balance sheet, the weak points become more obvious, and are yet more insistent when we recall our potential and proved capacity to control disease among the large masses of troops engaged in the late war. Obviously the hygiene administration of disciplined groups was easier than the corresponding administration of a general community; but, nevertheless, the principle is there. It is unity of administration which we must have. Standardisation of method, routine—call it what we will—but the sanitary and health service of this country must be made more and more to approximate to that smooth running uniform system of which the war showed us the value.

The vastness of the educative campaign required, not only among the people but the professions, young and old, is not a little difficult to comprehend; but, as Colonel Firth aptly says, though it is too early to see the effects of the newly formed Ministry of Health, judging by its utterances, its actions promise to be based on sound and comprehensive principles. He welcomes it as a great advance and the harbinger of great things to come, if only as a co-ordinator, director, developer, and unifier of forces which hitherto have overlapped or worked independently of each other. He foresees that it will be more than that, but the problem which it faces is complex to a degree, and the measure of its success will be judged by its organisation of an ordered and systematic attack on the strongholds of preventible disease, particularly that mass of crippling invalidism which is steadily undermining the capacity and efficiency of the masses. The essential partners in this attack are the public and the profession of medicine, or, what is the same thing, the health of the community depends mainly on three factors—namely, the good mother, the good general practitioner, and the good health officer.

